# Molecular Classifications in Gastric Cancer: A Call for Interdisciplinary Collaboration

**DOI:** 10.3390/ijms25052649

**Published:** 2024-02-24

**Authors:** Cristina Díaz del Arco, María Jesús Fernández Aceñero, Luis Ortega Medina

**Affiliations:** 1 Department of Legal Medicine, Psychiatry and Pathology, School of Medicine, Complutense University of Madrid, 28040 Madrid, Spain; jmariajesus.fernandez@salud.madrid.org (M.J.F.A.); luis.ortega@salud.madrid.org (L.O.M.); 2 Department of Pathology, Hospital Clínico San Carlos, Health Research Institute of the Hospital Clínico San Carlos (IdISSC), 28040 Madrid, Spain

**Keywords:** gastric cancer, molecular, classification, TP53, mesenchymal, microsatellite instability, tumor mutational burden, immune, prognosis

## Abstract

Gastric cancer (GC) is a heterogeneous disease, often diagnosed at advanced stages, with a 5-year survival rate of approximately 20%. Despite notable technological advancements in cancer research over the past decades, their impact on GC management and outcomes has been limited. Numerous molecular alterations have been identified in GC, leading to various molecular classifications, such as those developed by The Cancer Genome Atlas (TCGA) and the Asian Cancer Research Group (ACRG). Other authors have proposed alternative perspectives, including immune, proteomic, or epigenetic-based classifications. However, molecular stratification has not yet transitioned into clinical practice for GC, and little attention has been paid to alternative molecular classifications. In this review, we explore diverse molecular classifications in GC from a practical point of view, emphasizing their relationships with clinicopathological factors, prognosis, and therapeutic approaches. We have focused on classifications beyond those of TCGA and the ACRG, which have been less extensively reviewed previously. Additionally, we discuss the challenges that must be overcome to ensure their impact on patient treatment and prognosis. This review aims to serve as a practical framework to understand the molecular landscape of GC, facilitate the development of consensus molecular categories, and guide the design of innovative molecular studies in the field.

## 1. Introduction

Gastric cancer (GC) ranks as the fifth most common cancer worldwide, and is the third leading cause of cancer-related deaths. It is an aggressive disease, often diagnosed at advanced stages, with a 5-year survival rate of less than 30% [1,2].

In terms of classification, GC is a heterogeneous disease with multiple clinical, histological, and molecular variables influencing disease presentation and patient prognosis [3,4]. Geographical differences have been observed between Asian and Western countries, with GC being more prevalent in Asian regions. In fact, some high-incidence countries have implemented screening strategies that have improved early detection and patient outcomes [5,6,7]. Furthermore, geographic variations related to clinical, histological, prognostic, surgical, and treatment response factors have been noted [8,9,10].

Clinically, GC can be divided into proximal and distal types, each with distinct epidemiological characteristics. Proximal GC is associated with obesity, gastroesophageal reflux, and Barrett’s esophagus, while the more prevalent distal type is linked to *Helicobacter pylori* infections, the male gender, smoking, and dietary habits [1,11,12].

From a macroscopic perspective, GC can be classified using the Paris classification for superficial lesions, the Borrmann classification for advanced GC (stage pT2 or higher), or the Japanese Society of Endoscopy classification, encompassing both early and advanced GC [13,14,15].

With respect to histological features, notable classifications include the Laurén and the World Health Organization (WHO) systems [16,17]. Laurén’s classification, established in 1965 as a histoclinical classification, categorizes GC into intestinal and diffuse types. Intestinal GC forms tubules and may include papillary or solid structures, occurs in older patients, and is associated with *H. pylori* infection and environmental factors. It develops through the carcinogenic process of chronic gastritis—intestinal metaplasia—dysplasia [18]. In contrast, diffuse GC is composed of loosely cohesive cells, potentially displaying signet ring morphology, appears in younger patients, and is induced by active inflammation or genetic factors. Previous studies have shown that this classification correlates with patient prognosis, treatment response, and the molecular characteristics of GC [8]. On the other hand, the WHO classification is more complex, morphology-based, and identifies the following four main types of GC: tubular, papillary, poorly cohesive, and mucinous [19]. This classification has shown a lower correlation with non-histological factors [20]. It should be noted that both classifications establish a "mixed" subtype.

Regarding GC treatment, surgery remains the only curative option for GC [21]. Endoscopic techniques can be employed in early stages, while more advanced stages, prevalent in Western countries, require a total or subtotal gastrectomy with lymphadenectomy [22]. For non-surgical patients, chemotherapy is the main therapeutic approach [23]. Approved targeted drugs include antiangiogenics (anti-VEGFR-2) and anti-HER2 agents [24,25]. Additionally, the approval of pembrolizumab for solid tumors with high microsatellite instability (MSI-H) or mismatch repair deficiency (dMMR) included GC cases [26,27]. The indication for immunotherapy also depends on PD-L1 expression or the tumor mutational burden (TMB) [28]. Therefore, the only established and broadly available biomarkers for GC treatment are *HER2* amplification, MSI-H, and PD-L1 expression [29]. The therapeutic arsenal for GC is limited when compared to other tumor types, and current therapies have not significantly improved patient prognosis [30,31].

In terms of molecular characteristics, technological advancements in recent years have allowed the identification of multiple molecular alterations in various types of tumors [32]. Among these, alterations with prognostic or therapeutic value have significantly impacted clinical practice in tumors such as lung or breast cancer, enabling personalized treatment, improving patient outcomes, and reducing the side effects associated with conventional treatment [33,34].

In GC, multiple studies have analyzed its genetic, epigenetic, transcriptomic, proteomic, or metabolomic profiles, revealing numerous molecular changes and dysregulated pathways, some of which carry prognostic and/or therapeutic significance [35,36,37,38,39,40,41]. The synthesis of this information has given rise to several molecular classifications, with notable examples being those published by The Cancer Genome Atlas (TCGA) and the Asian Cancer Research Group (ACRG) [42,43]. Despite these efforts, the practical impact of these classifications on clinical practice remains limited, primarily due to the complexity of their implementation. Beyond these pivotal studies, various authors have proposed alternative molecular classifications of GC that require external validation in other cohorts and the identification of surrogate markers for their application. Consequently, there is an urgent need to reach a consensus on molecular categories, establish easily detectable subgroups, and identify optimal surrogate markers for each molecular subtype.

In this review, our objective is to revisit diverse molecular classifications published in GC from a practical standpoint. We aim to highlight the correlation between these systems, the molecular alterations specific to each subtype, and their associations with clinicopathological, prognostic, and therapeutic factors. We have selected the most significant classifications, along with others offering alternative perspectives, in order to provide a comprehensive overview of GC heterogeneity. Furthermore, we discuss the challenges that must be overcome to ensure these classifications have an impact on clinical practice, ultimately improving patient treatment and prognosis.

## 2. Gastric Cancer Characterization, Prognosis, and Management in the Molecular Era

As previously mentioned, recent technological advancements have propelled cancer research into the molecular era. Comprehensive genetic, transcriptomic, and proteomic analyses are now possible, resulting in vast databases of molecular changes, including mutations, copy number variants, epigenetic alterations, gene expression profiles, or disrupted pathways across various tumor types. This wealth of information has enabled the identification of molecular alterations with prognostic and therapeutic significance. Prognostic alterations allow for personalized patient management, improving the cost-effectiveness of treatment. Meanwhile, predictive molecular alterations have transformed cancer treatment from a generic approach to an individualized approach. Targeted drugs have enhanced patient prognosis, often with fewer side effects and better tolerance than conventional chemotherapy [44].

### 2.1. The Molecular Era: Recent Advances in Molecular Techniques

Among the technological advances that have impacted the molecular characterization of cancer in the last decade, microarrays and second- or next-generation sequencing platforms (NGS) stand out. Microarrays allow the detection of molecular alterations at the DNA, RNA, or protein level [45,46,47]. They are primarily applied in research studies, although some commercial microarray-based platforms are used in clinical routine, mainly in breast cancer [48,49]. NGS techniques, which have been implemented in clinical practices in institutions worldwide, are typically employed for DNA sequencing. They facilitate the simultaneous analysis of multiple samples, either at the whole genome or whole exome level, or through the utilization of targeted panels containing dozens of genes of interest. These techniques have been refined, automated, and modified to allow for the analysis of RNA or epigenetic alterations. In GC, NGS and microarrays have played a pivotal role in elucidating the landscape of molecular alterations [50,51,52,53]. However, in the daily practice of GC, these techniques do not present significant applications because the necessary biomarkers are currently analyzed using immunohistochemistry (IHC) and in situ hybridization methods. NGS could be useful for determining the TMB, or as a complementary technique for assessing MSI status [54,55,56,57].

In the early 2010s, third-generation sequencing techniques emerged, enabling sequencing at a single-molecule level. Despite their potential, these techniques have not been integrated into clinical practice, and their utilization in research studies remains limited. Advantages over second-generation sequencing methods include the fact that they do not require sample pre-amplification and can read longer fragments of DNA, but the error rate is generally higher (10–15%) [58,59,60,61].

Lastly, another interesting molecular approach that has garnered attention in recent years is single-cell sequencing (SCS), which, using second- or third-generation methodologies, enables the analysis of DNA, RNA, or methylome at the single-cell level [62,63,64,65]. Its main advantage lies in its ability to scrutinize the molecular profile of cell subclones, thereby offering significant potential for evaluating tumor heterogeneity, refining the personalization of patient management, and enhancing the monitoring of treatment response and resistance detection [66,67]. Furthermore, SCS requires a small sample size, thus making it suitable for analyzing circulating tumor cells in liquid biopsy specimens [68]. However, these techniques have not yet been implemented in clinical routine and require technical refinement, standardization, and cost reduction to have a practical impact [69,70]. In GC, research in this area is in its early stages, but promising results have been obtained [71,72,73].

### 2.2. Main Molecular Alterations in Gastric Cancer

Multiple molecular alterations and dysregulated pathways have been identified in GC. Notably, mutations in the *TP53* and *CDH1* genes are prominent [74,75]. *TP53* mutation is the most common in GC, occurring in over 50% of cases, and is often associated with chromosomal instability and an increased expression of cell-cycle progression genes [76,77]. While the *TP53* mutation has been correlated with a worse prognosis in other tumors, its significance in GC remains unclear [75,78,79,80]. This uncertainty may stem from the specific impact of different mutations on the function of the p53 protein, concomitant molecular alterations, or treatment effects [75,81]. Additionally, most studies have focused on the p53 protein rather than the gene, with some exceptions [75,77,82,83]. As for *CDH1*, it encodes for E-cadherin, a transmembrane glycoprotein responsible for maintaining cell–cell adhesion [84]. Germline mutations in *CDH1* are associated with hereditary diffuse GC syndrome, which increases the risk of diffuse GC and lobular breast carcinoma [85]. In sporadic GC cases, mutations and the abnormal methylation of CDH1 are predominantly found in diffuse GC [86,87]. Other key mutations in GC include those within the *ARID1A*, *PIK3CA*, or *BRCA2* genes [88,89,90].

Regarding copy number alterations, the amplification of genes involved in tyrosine kinase receptor pathways, such as *FGFR2*, *HER2*, *EGFR*, or *MET*, stand out [91,92,93,94]. Among these genes, *HER2* amplification has significant clinical implications, serving as an indication for treatment with trastuzumab in advanced HER2-positive GC patients [95,96]. *HER2* amplification occurs in 10–15% of patients with advanced GC, with a higher prevalence in intestinal-type GC and a lower prevalence in diffuse GC [97,98,99]. The most frequent copy number variation is the amplification of *FGFR2*, which is observed in 15% of patients and associated with high-grade tumors and a poorer prognosis [93].

Finally, the main dysregulated pathways in GC include those related to genome integrity, cell adhesion, chromatin remodeling, cell motility and cytoskeletal structure, Wnt signaling, and tyrosine kinase receptors [74].

### 2.3. Current Treatment of Gastric Cancer

As for the management of GC, surgery remains the only curative option, and most resectable tumors are treated with total or subtotal gastrectomy associated with D2 lymphadenectomy. Early stage tumors meeting certain criteria may undergo endoscopic procedures, such as endoscopic mucosal resection or endoscopic submucosal dissection [100]. The assessment of tumor depth, size, grade, and the presence of ulceration is crucial to determine the suitability of these techniques [101].

Surgery for GC typically forms part of a multimodal treatment, with the two following options, depending on the context: surgery followed by adjuvant chemotherapy or perioperative therapy. Regarding the surgical procedure, according to the European Society for Medical Oncology (ESMO) guidelines, T1 tumors can be treated with partial gastrectomy and D1 lymphadenectomy, while for IB-III disease, total or subtotal gastrectomy with D2 lymphadenectomy is recommended [101]. Perioperative chemotherapy has become the standard of care, supported by findings from clinical trials which have been conducted since the 2000s, demonstrating a survival benefit for patients undergoing this approach [102,103,104]. ESMO guidelines advocate for the pre- and post-operative administration of FLOT regimen (5-FU, leucovorin, oxaliplatin, and docetaxel) in patients who can tolerate it [101]. The choice of the chemotherapy regimen may vary depending on the guideline, and the role of radiotherapy as an adjunct is still under investigation [105,106,107].

The main innovations in the surgical treatment of GC include the use of laparoscopy, which has been shown to be non-inferior to open surgery in both Asian and Western countries, and robot-assisted gastrectomy [108,109,110,111].

Regarding non-surgical cases, it is worth noting that, despite the detection of numerous molecular alterations and the development of multiple molecular classifications in GC, the clinical application of this information lags behind other cancers. For instance, breast cancer has successfully integrated molecular classification into daily practice, surpassing the practical impact of traditional histological features. In lung cancer, multiple targetable alterations have been identified, leading to recommendations for testing as many as nine molecular biomarkers and PD-L1 expression in all adenocarcinomas and in squamous cell carcinomas that meet certain criteria [112]. As a final example, in endometrial cancer, molecular and histopathological features have been integrated to develop a new FIGO staging system with prognostic and therapeutic value, which has been in effect since 2023 [113].

Contrastingly, in unresectable GC, the main therapeutic approach continues to be conventional chemotherapy, typically involving a platinum-fluoropyrimidine doublet [114]. Nonetheless, many patients develop resistance to this treatment, which often leads to adverse effects [115,116,117]. 

The administration of targeted therapy has the potential to enhance the specificity and efficacy of oncological treatment, while mitigating adverse effects [118]. As drawbacks, the effectiveness of these therapies is heavily reliant on the molecular profile of the tumor at a given time, and they are not entirely devoid of toxicity [44,119]. According to the latest National Comprehensive Cancer Network guidelines, the main targeted therapies approved for advanced GC include anti-HER2 agents (trastuzumab and fam-trastuzumab deruxtecan-nxki), anti-VEGFR-2 agents (ramucirumab), and immunotherapy (nivolumab, pembrolizumab and dostarlimab-gxly) [120]. Anti-HER2 therapy is indicated in *HER2*-amplified GC, and immunotherapy may be indicated in cases with MSI-H, PD-L1 overexpression, or a high TMB [120]. However, the latest ESMO guidelines only include PD-L1 expression and MSI-H as indications for immunotherapy [101]. Lastly, tumors with *NTRK1*, *NTRK2*, or *NTRK3* gene fusions may be treated with entrectinib and larotrectinib, although such cases are exceptionally rare in GC, with only one case published so far [121].

### 2.4. Gastric Cancer: Therapeutic Advances and Challenges

Early GC has demonstrated favorable outcomes for decades, with survival rates of over 90% with surgical treatment [122,123]. However, in Western countries, the lack of widespread screening techniques coupled with mild and nonspecific symptoms has lead to over 80% of patients being diagnosed at advanced stages [124]. Despite advancements in molecular biology and personalized therapy, the prognosis for advanced GC has seen limited improvement [125]. Even in resectable cases, the recurrence rates range from 14–80%, often exceeding 40% within the first years following surgery [126,127]. The addition of neoadjuvant therapy in surgical cases has slightly improved patient prognosis, but studies report recurrence rates exceeding 30% [128,129,130]. Unresectable cases present a dismal prognosis, with median overall survival rates ranging from 11 to 14 months, and 5-year survival rates of less than 30% [131,132,133,134]. Notably, patients eligible for targeted therapy or immunotherapy exhibit significantly higher survival rates overall [135,136]. However, numerous authors highlight the need to refine patient selection for these treatments, enhance drug efficacy, identify new therapeutic targets, and overcome treatment resistance [137,138,139,140].

The modest impact of the aforementioned advancements on the prognosis and management of advanced GC and the scarcity of therapeutic targets could be due to the heterogeneity that characterizes this tumor, both phenotypically and molecularly [3,4,141]. This heterogeneity is also evident at the intratumoral and tumor microenvironmental levels, as demonstrated by recent single-cell studies [142,143]. Additionally, molecular heterogeneity exists among primary tumors, lymph node metastases, and distant metastatic sites [144,145,146]. Understanding spatial and temporal heterogeneity, both phenotypically and molecularly, at primary and metastatic sites holds promise for improving prognosis and treatment outcomes for GC patients.

New potential treatment strategies for GC encompass perioperative targeted therapy or immunotherapy, personalized treatment guided by molecular tumor characterization, the utilization of trastuzumab conjugates, and the development of new anti-HER2 agents. Additionally, ongoing studies are investigating novel therapeutic approaches, such as Claudin 18.2 targeted therapy or *FGFR*, *MET*, and *EGFR* inhibitors [147,148].

In summary, these circumstances highlight the need for enhancing patient stratification in both clinical trials and practice. Additionally, identifying new biomarkers and improving the currently available drugs is crucial to expand the range and effectiveness of targeted therapies for GC and translate the progress seen in other tumors to GC.

## 3. Molecular Classifications in Gastric Cancer

As mentioned earlier, the most significant molecular systems published in GC are those of TCGA and the ACRG, which have been extensively analyzed in previous studies [74,149,150,151,152,153,154]. Nevertheless, various alternative classifications have emerged, either utilizing independent cohorts or drawing from existing databases, such as those of TCGA, the ACRG, or the Gene Expression Omnibus (GEO) repository. These alternative classifications introduce diverse perspectives, encompassing metabolic, immune, mutational burden, proteomic, or epigenetic approaches, among others. The methodology and key findings of each classification are presented in this section.

### 3.1. The Cancer Genome Atlas (2014) [42]

Researchers from TCGA analyzed tumor and non-tumor samples from 295 patients, conducting array-based somatic copy number analysis, whole-exome sequencing, array-based DNA methylation profiling, messenger RNA sequencing, microRNA sequencing, and reverse-phase protein array [42]. Subsequently, they performed the unsupervised clustering of the information obtained from each molecular method and integrated the results, leading to the following four molecular groups: tumors positive for Epstein–Barr virus (EBV), microsatellite instability (MSI), genomically stable (GS), and GC with chromosomal instability (CIN). 

The MSI type exhibited a high mutation rate, affecting genes such as *PIK3CA*, *ERBB2*, *ERBB3*, or *EGFR*. Additionally, gene promoter methylation was observed, with the frequent methylation of the *MLH1* gene promoter. These tumors were not characterized by the presence of the V600E *BRAF* mutation or the amplification of tyrosine kinase receptor genes. MSI tumors appeared more frequently in women, older patients, and distal stomach

EBV-positive GC mainly presented mutations in *PI3CA* (80%), *ARID1A* or *BCOR*, *HER2* amplification, *JAK2* amplification, and the overexpression of PD-L1 and PD-L2. This group was defined by extensive DNA methylation without *MLH1* hypermethylation, and showed enrichment in immune-related pathways. EBV-positive GC occurred in younger individuals, males, and in the gastric antrum.

GS tumors exhibited mutations in *RHOA* (15%), *ARID1A*, and *CDH2*, as well as *CLDN18-ARHGAP26* fusion. Interestingly, *RHOA* mutation and *CLDN18-ARHGAP26* fusion were mutually exclusive. These tumors were enriched in pathways related to cell adhesion and angiogenesis. They appeared mainly in the gastric antrum of younger patients and were more frequently of the diffuse type.

Finally, CIN-type GC was characterized by the amplification of tyrosine kinase receptor genes (such as *VEGF*, *FGFR2*, or *HER2*), cell cycle-related genes (*CCNE1*, *CCND2*, or *CDK6*), and mutations in genes such as *TP53* (71%), *ARID1A*, *KRAS*, *PIK3CA*, *ERBB3*, *PTEN*, or *HLA-B*. These tumors were predominantly of the intestinal type, and were mainly located in the esophagogastric junction or gastric cardia.

### 3.2. Asian Cancer Research Group (2015) [43]

**Figure 1:**
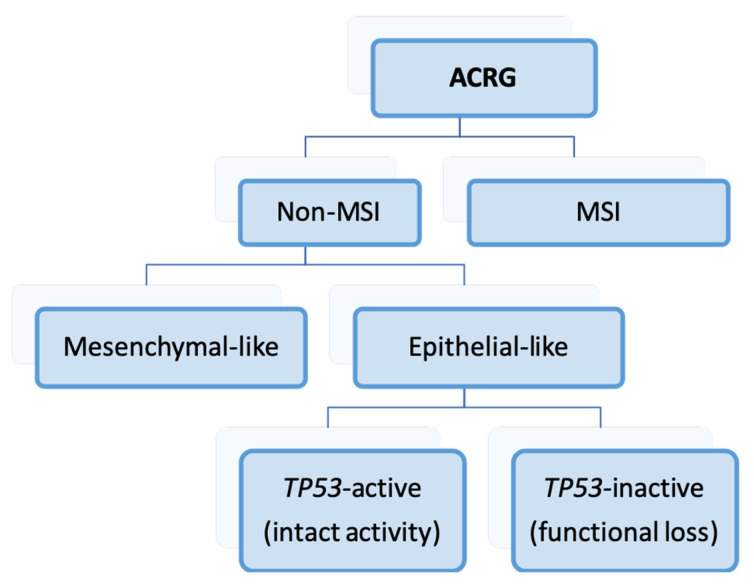
Algorithm for the classification of cases, according to the Asian Cancer Research Group system [43]. ACRG: Asian Cancer Research Group; MSI: microsatellite instability.

The ACRG studied GC samples from 251 patients, utilizing gene expression profiling, genome-wide copy number microarrays, and targeted gene sequencing [43]. The authors defined the following four molecular subtypes, subsequently validating them in various GC patient cohorts: MSI, *TP53*-active, *TP53*-inactive, and mesenchymal-like. To determine these categories, a diagnostic algorithm is required, as seen in Figure 1. Notably, the *TP53* status was determined using a two-gene *TP53*-activity signature (*CDKN1A* and *MDM2*).

The MSI subtype exhibited a high mutation rate, with alterations in the genes of the PIK3CA-AKT-mTOR pathway, *KRAS*, *ALK*, or *ARID1A*. It also displayed extensive DNA methylation and a loss of *MLH1* expression. MSI GC was predominantly intestinal, more commonly located in the gastric antrum, and had the most favorable prognosis. Mesenchymal-like GC was associated with epithelial–mesenchymal transition (EMT), featured the loss of *CDH1* expression and low mutation events, appeared in younger patients at advanced stages, and 80% of them were of the diffuse type. It represented the molecular type with the poorest prognosis. Conversely, the *TP53*-inactive subtype was characterized by the amplification of genes such as *HER2*, *EGFR*, *CCNE1*, *CCND1*, *MDM2*, *ROBO2*, *GATA6*, or *MYC*. *TP53* mutations were observed in 60% of *TP53*-inactive cases, and those patients showed an intermediate prognosis. Finally, *TP53*-active GC presented mutations in *PIK3CA*, *ARID1A*, *KRAS*, *SMAD4*, or *APD*, with *TP53* mutations identified in 23.7% of cases. This subtype also had an intermediate prognosis, albeit a slightly better one than *TP53*-inactive GC.

### 3.3. Intrinsic Subtypes (2011) [155]

Tan et al. cultivated and analyzed 37 GC cell lines from commercial providers and collaborators, utilizing unsupervised clustering techniques to identify two intrinsic subtypes of GC [155]. These subtypes were subsequently validated in primary tumors from four independent cohorts, encompassing 521 patients from Singapore, Australia, and South Korea. It is important to note that using cell lines does not allow for the assessment of the tumor microenvironment (including stroma, blood vessels, or immune cells). Therefore, this approach presents both advantages and disadvantages. 

The researchers established the following two categories of GC: G-INT and G-DIF [155]. G-INT GC (49.35–57%) exhibited the upregulation of genes related to carbohydrate and protein metabolism (*FUT2*) and cell adhesion (*LGALS4*, *CDH17*). G-DIF GC (43–50.65%) showed the enrichment of genes related to fatty acid metabolism (*ELOVL5*) and cell proliferation (*AURKB*). These categories can be determined by gene expression profiling techniques (analyzing 171 genes with differential expression between the types) or conducting IHC stains to analyze LGALS4 and CDH17 expression. According to the IHC classification, a tumor is considered G-INT if both markers are positive, and G-DIF if neither is positive. Positivity is based on staining intensity (CDH17 > 1+ and LGALS4 > 2+). Cases with only one positive stain are deemed equivocal. The authors emphasize that further studies should validate these markers or search for others with better performances as surrogates for their classification.

#### Relationship with Clinicopathological, Prognostic, and Therapeutic Variables

A significant relationship was observed between Tan’s intrinsic subtypes, the Laurén classification (64% concordance), and the histological grade [155]. Regarding its prognostic role, this molecular classification was identified as an independent prognostic factor, with G-DIF tumors showing worse survival outcomes [155]. In contrast, the Laurén classification did not exhibit significant or independent prognostic value. Notably, patients with G-INT diffuse-type tumors demonstrated better survival than those with G-DIF intestinal-type GC. 

Concerning therapeutic implications, in vivo and in vitro studies indicated that G-INT tumors were more sensitive to 5-FU and oxaliplatin, while G-DIF tumors were more responsive to cisplatin [155]. Additionally, in two patient cohorts, a significant interaction was observed between intrinsic subtypes and the benefit of 5-FU-based chemoradiation. Patients with G-INT tumors exhibited a significant survival benefit with 5-FU treatment, whereas patients with G-DIF tumors showed no differences in the stage-adjusted hazard ratio of death due to cancer between the 5-FU treated and untreated subgroups.

### 3.4. Lei’s Classification (2013) [156]

Lei et al. analyzed the expression profiles of 248 GC cases from Singapore, and determined intrinsic subtypes using unsupervised clustering [156]. Subsequently, the subtypes were validated in an independent set of 70 tumors from Australian patients.

**Figure 2:**
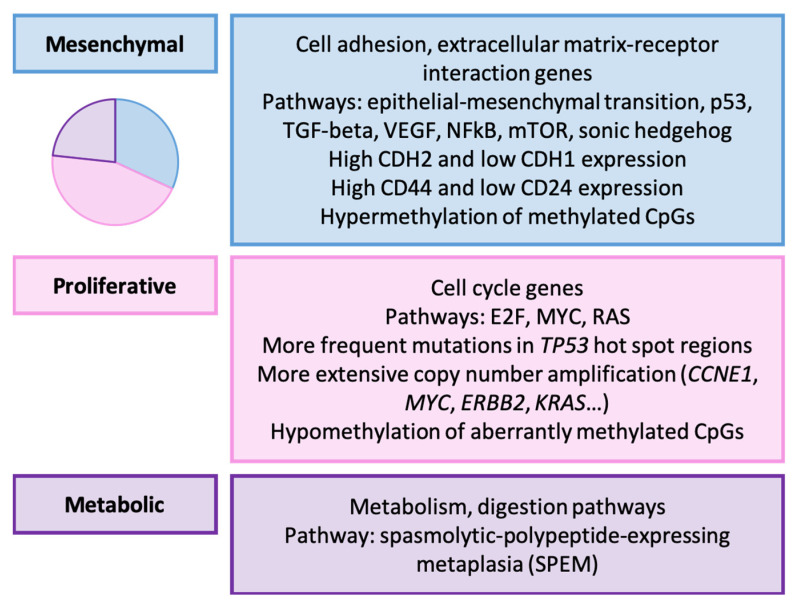
Lei’s classification. Molecular characteristics of GC types [156].

Three GC groups were established as follows: mesenchymal (31.9%), proliferative (44.8%), and metabolic (23.3%). Their molecular characteristics are presented in Figure 2.

#### Relationship with Clinicopathological, Prognostic, and Therapeutic Variables

This classification was significantly associated with Laurén type, histological grade, and GC location [156]. The mesenchymal subtype was enriched in diffuse-type GCs and high-grade tumors, while the proliferative subtype had the highest percentage of intestinal-type and low-grade GCs. In terms of location, the middle stomach had the highest occurrence of tumors, especially in the metabolic type. Mesenchymal and proliferative GCs were more prevalent in the lower and upper stomach, respectively.

Survival analysis did not reveal significant or independent differences in cancer-specific survival (CSS) or disease-free survival (DFS) between molecular categories. However, it was noted that patients with metabolic GC benefited more from 5-FU treatment compared to other subtypes, a finding corroborated by the researchers in cell line experiments. Additionally, mesenchymal GC cell lines exhibited a heightened sensitivity to compounds targeting the PIK3CA-AKT-mTOR pathway.

### 3.5. Wang’s Classification (2021) [157]

Wang et al. conducted whole-exome sequencing of 70 GC samples from Chinese patients, each paired with normal mucosa. They validated their classification in 23 cases from an independent cohort [157]. To establish molecular categories, the authors applied unsupervised clustering, combining genomic features (mutational signatures, copy number variations, neoantigens, clonality, and others), clinicopathological features, metastatic patterns, and overall survival (OS). PD-L1 staining by IHC was also performed.

**Figure 3:**
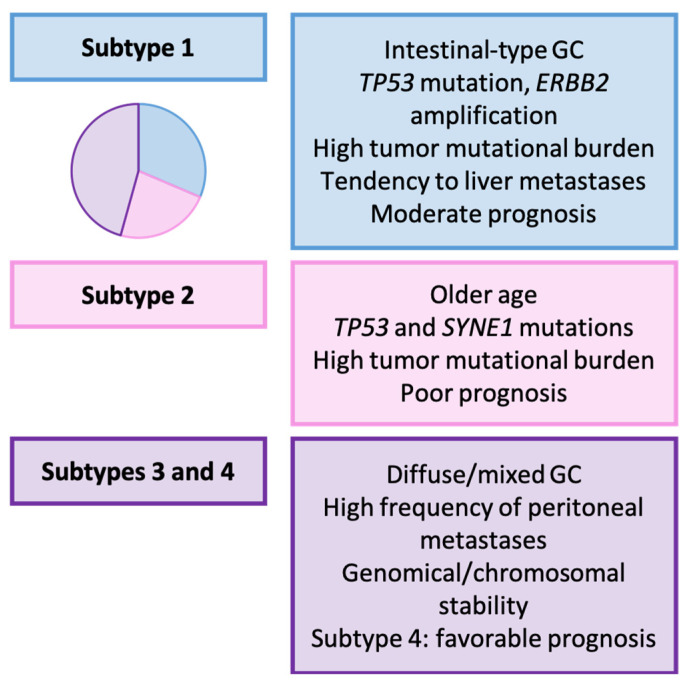
Wang’s classification (2021). Clinicopathological and molecular features of GC types [157].

Tumors were categorized into subtype 1 (31.4%), subtype 2 (22.9%), subtype 3 (17.1%), and subtype 4 (28.6%). The main characteristics of each subtype are summarized in Figure 3. Subtypes 1 and 2 primarily exhibited mutations in *FAT4*, *LRP1B*, and *TP53*. Additionally, subtype 2 showed more frequent alterations in *SYNE1*, *CSMD1*, *CSMD3*, and *SPTA1*, while subtype 1 displayed a higher frequency *PIEZO1* alterations. Subtypes 3 and 4 showed a lower frequency of gene mutations, with *ARID1A*, *TP53* (though to a lesser extent), *SYNE1*, and *SPTA1* being the most frequently mutated genes. Copy number variations were enriched in subtype 1, with amplifications of genes such as *ERBB2* or *HSP90AB1*.

#### Relationship with Clinicopathological, Prognostic, and Therapeutic Variables

The authors observed a significant relationship between their classification, Laurén types, TCGA molecular classification, the first site of metastasis, and the TMB [157].

Subtypes 1 and 2 were predominantly of the intestinal type, while subtypes 3 and 4 were more frequently of the diffuse type. Regarding the association with TCGA system, the majority of subtype 1 tumors were classified as CIN (>90%), whereas 50% of subtype 2 GCs were CIN, and 50% were GS. Most subtype 3 and 4 tumors were GS tumors (83.3% and 75%, respectively). In terms of TMB, subtypes 1 and 2 showed a higher TMB (36.4% and 50% with TMB > 5, respectively) and increased PD-L1 expression.

Concerning patient prognosis, subtype 4 GC exhibited superior OS, followed by subtype 1, while subtype 2 and 3 GC demonstrated the least favorable OS. This molecular classification not only provided prognostic insights, but also maintained independent prognostic value.

Lastly, subtype 1 GC showed a higher likelihood of spreading to the liver when compared to other subtypes, while subtypes 3 and 4 displayed a greater incidence of peritoneal involvement. This observation carries potential importance for stratifying the risk of peritoneal involvement, indicating the possible effectiveness of intraperitoneal chemotherapy in high-risk patients.

### 3.6. Shah’s Classification (2011) [158]

Shah et al. investigated the expression profiles of 36 GC samples from Western patients through supervised analysis [158]. Prior to molecular studies, drawing on the previous literature, the authors determined the following three GC subtypes: proximal non-diffuse (33.3%), diffuse (27.7%), and distal non-diffuse (38.8%).

Proximal non-diffuse tumors predominantly occupied the gastric cardia (>80% of the tumor bulk), with potential extension to the gastroesophageal junction or distal esophagus. These tumors were intestinal or mixed, and ranged from well to poorly differentiated. Adjacent areas displayed glandular dysplasia or carcinoma in situ, linked to chronic inflammation without atrophy. In contrast, diffuse tumors were entirely diffuse, with minimal extracellular mucin, and could be located anywhere in the stomach. Finally, distal non-diffuse GC primarily comprised moderately differentiated intestinal tumors with minor high-grade components, associated with chronic gastritis and intestinal metaplasia. Similar to proximal non-diffuse tumors, they exhibited a spectrum of dysplasia and/or carcinoma in situ in nearby regions.

Following subtype determination, the researchers conducted gene expression profiling, comparing tumor samples with normal stomach samples in order to discern molecular characteristics distinguishing each subtype (Table 1).

Their findings indicated a general upregulation of cancer-related genes in all tumors, including those related to the cell cycle, cell proliferation, adhesion, platelet-derived growth factor binding, and EGF-domain pathways. Concurrently, there was a downregulation of genes related to lipid metabolism or digestion pathways.

Compared to normal mucosa, proximal and distal non-diffuse GC were enriched in cell cycle, mitosis, and p53 pathways, and exhibited the downregulation of digestion and drug metabolism pathways. The exploration of crucially altered pathways revealed the upregulation of glycogenesis and gluconeogenesis in these two subtypes. On the other hand, diffuse tumors did not show significantly upregulated pathways.

While the authors did not place significant emphasis on the prognostic or therapeutic implications of their classification, they noted that tumors with elevated expressions of PLA2G2A may exhibit a more favorable prognosis. Additionally, the reported association between the tumor location and the Laurén subtypes with the prevalence of *HER2* amplification adds potential clinical utility to this classification [159,160].

### 3.7. HOPE Classification (2022) [161]

**Figure 4:**
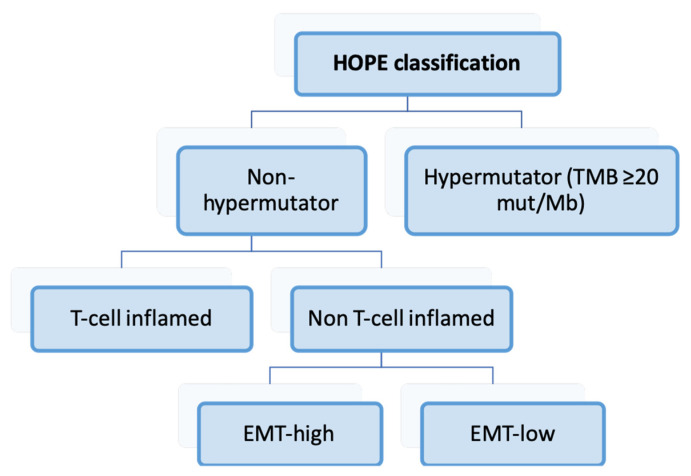
Algorithm for the categorization of cases according to the HOPE classification [161]. TMB: tumor mutational burden; EMT: epithelial-mesenchymal transition.

Furukawa et al. conducted whole-exome sequencing and gene expression analysis on 499 tumor samples from Japanese patients [161]. They established the four following distinct types of GC: hypermutators (10.8%, HMT), T-cell inflamed (33.5%, TCI), EMT-high (18.6%, EMTH), and EMT-low (37.1%, EMTL), employing the algorithm outlined in Figure 4.

In relation to the TCGA classification, all HMT cases exhibited MSI, although MSI tumors were also observed in the remaining subgroups. EMTH and EMTL cases predominantly fell into the GS and CIN categories, according to TCGA. Similarly, these molecular types aligned mainly with the mesenchymal-like and *TP53*-inactive categories, respectively, according to the ACRG. 

#### Relationship with Clinicopathological, Prognostic, and Therapeutic Variables

**Figure 5:**
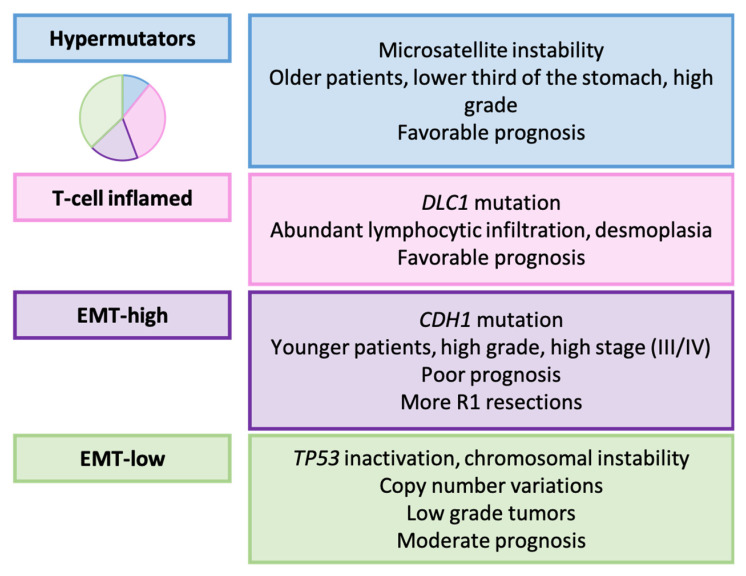
HOPE classification. Clinicopathological and molecular characteristics of GC types [161].

The molecular classification demonstrated significant associations with the patient’s age, GC histology, location, and the pathological stage, as summarized in Figure 5. Grouping HMT and TCI GCs revealed significant differences in prognosis, with these two types having the best prognosis, followed by EMTL and EMTH GC. The EMTH category emerged as an independent prognosticator in multivariate analysis.

Non-EMTH subgroups seemed to benefit from adjuvant chemotherapy, while HMT or TCI tumors could potentially benefit from immunotherapeutic strategies. The authors suggested that EMTH tumors might respond positively to drugs targeting mesenchymal cell-specific proteins.

### 3.8. Wang’s Classification (2014) [162]

**Figure 6:**
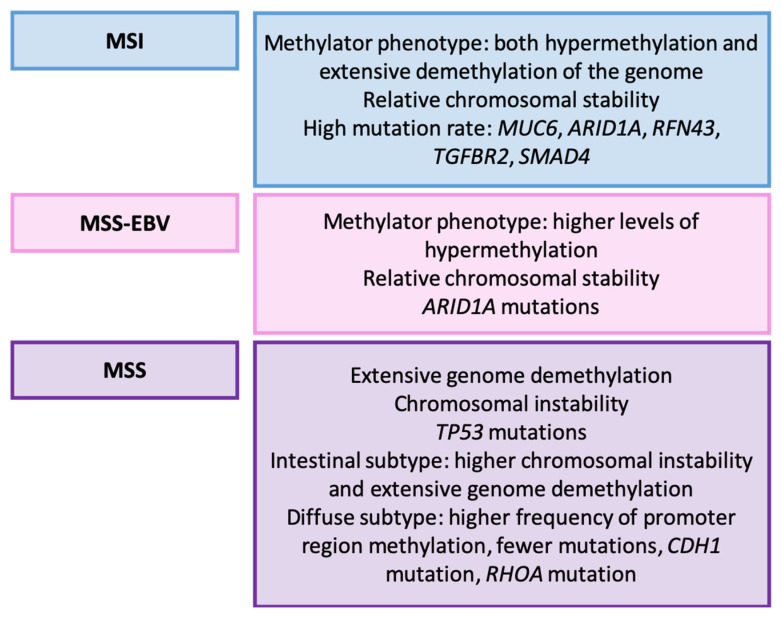
Wang’s classification (2014). Molecular features of GC types [162]. MSI: microsatellite instability; MSS-EBV: stable Epstein-Barr virus-positive; MSS: stable.

Wang et al. conducted a comprehensive study involving whole-genome sequencing, DNA copy number analysis, gene expression profiling, and methylation profiling of 100 paired tumor and non-tumor samples from gastrectomy specimens, validating their findings in 99 paired GC and non-GC samples [162]. They identified the three following types of GC: MSI, stable associated with EBV, and stable not associated with EBV. Their key characteristics are outlined in Figure 6.

#### Relationship with Clinicopathological, Prognostic, and Therapeutic Variables

A significant relationship was observed between Laurén types and the detected molecular alterations [162]. Additionally, *RHOA* mutation showed a significant correlation with the histological grade, tumor location, and *TP53* mutation. Although the researchers did not explicitly determine the prognostic value of their classification, prior studies have indicated that MSI and EBV tumors generally exhibit a better prognosis. In terms of therapeutic value, several detected alterations, including *RHOA* mutation, hold potential therapeutic significance [163].

### 3.9. Cheong’s Classification (2022) [164]

Cheong et al. conducted a comprehensive analysis, involving samples from 612 South Korean patients and data from TCGA, the ACRG, and other cohorts [164]. Applying a machine learning algorithm, they developed a GC-specific 32-gene signature, and performed unsupervised clustering to identify the following four molecular types of GC: group 1 (20.1%), group 2 (22.8%), group 3 (28.6%), and group 4 (28.6%).

**Figure 7:**
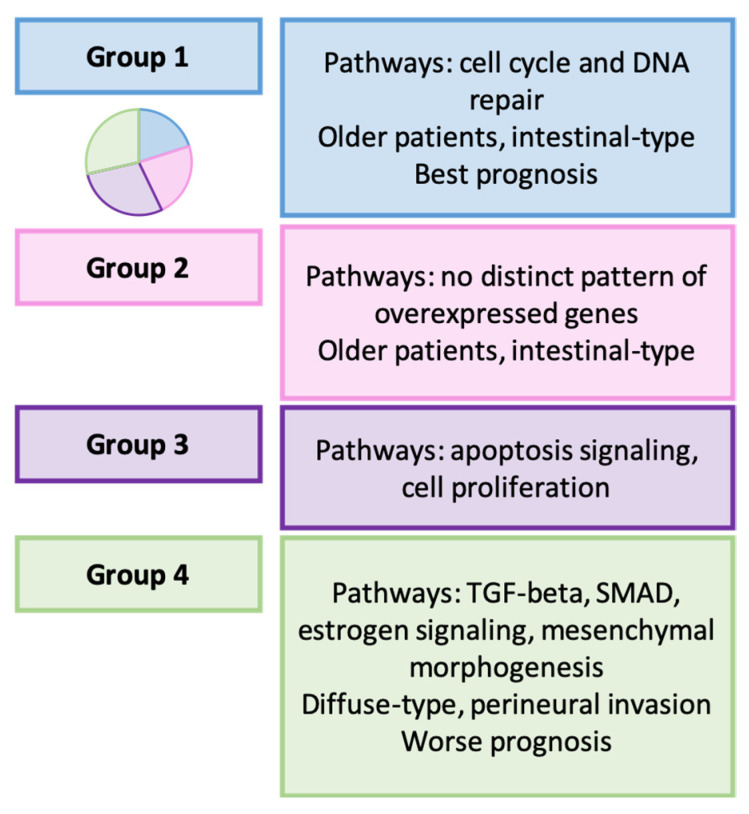
Cheong’s classification. Clinicopathological and molecular features of GC types [164].

The main characteristics of the four molecular groups are summarized in Figure 7. The authors did not find differences in the prevalence of MSI or EBV tumors between the groups. In a prior study, the same research group analyzed the gene expression profile of 1259 GC specimens, identifying the following five transcriptomic-based molecular subtypes related to patient prognosis: inflammatory, mesenchymal, intestinal, gastric, and mixed-stromal. In the current study, they observed that the mesenchymal type was enriched in group 4. Additionally, mesenchymal-like tumors according to the ACRG were more frequent in group 4, and MSI tumors were distributed among groups 2–4.

Concerning GC management, group 3 GC seemed to benefit from adjuvant chemotherapy, while in patients from group 1, this treatment could be potentially harmful. Notably, the response to pembrolizumab was superior in patients from groups 1 and 3.

### 3.10. Zhou et al. (2023): Functional Status-Based Classification [165]

**Figure 8:**
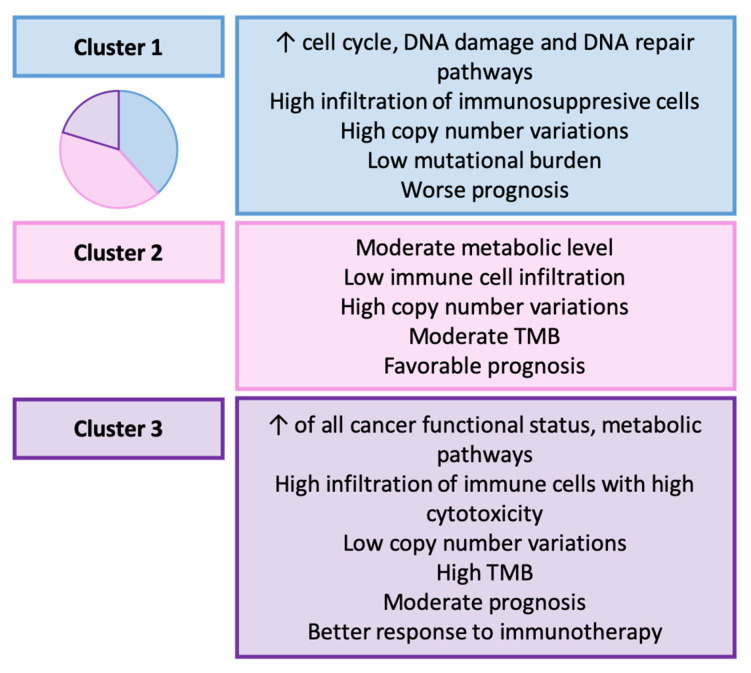
Zhou’s classification. Clinicopathological and molecular characteristics of GC types [165]. TMB: tumor mutational burden.

Zhou et al. examined gene expression profiles and clinical data from TCGA and GEO databases [165]. They calculated 14 cancer functional status scores and established 3 molecular subtypes of GC as follows: cluster 1 (38.4%), cluster 2 (41.3%), and cluster 3 (20.3%). The main features of these subtypes are presented in Figure 8. This classification holds the following prognostic and therapeutic implications: the investigators discovered that 5-FU, cisplatin, docetaxel, mitomycin C, and paclitaxel were more effective in cluster 1 GC, and this group of patients exhibited a worse prognosis. Additionally, they proposed multiple drugs with specific effects on each cluster.

### 3.11. Ye et al. (2022): Metabolism-Based Classification [166]

Ye et al. established subtypes similar to those in Lei’s classification, focusing on GC metabolism [156]. They conducted the unsupervised classification analysis of clinical and transcriptomic data from 161 cases retrieved from various databases, including Genomic Data Commons, GEO, and TCGA cohorts [166].

The authors identified the four following molecular subtypes: immune suppressed (C1), metabolic (C2), mesenchymal/immune exhausted (C3), and hypermutated (C4). Table 2 outlines the main characteristics of these four categories. C2 and C4 subtypes exhibited higher metabolic activity. Interestingly, there was a significant correlation between Ye’s classification and TCGA, Laurén, Lei’s, and WHO classifications. Types C2 and C3 resembled the metabolic and mesenchymal types from Lei’s classification, respectively. Concerning TCGA system, over 90% of C1 tumors were categorized as CIN. C2 tumors were predominantly CIN (64.7%) or GS (26.5%). C3 GC was mainly GS (47.1%) or CIN (40%), and G4 tumors were primarily CIN (39.8%) or MSI (37.5%). As shown in Table 2, Ye’s classification was also linked to treatment response and patient prognosis.

### 3.12. Li et al. (2021): Metabolism-Based Classification [167]

The authors analyzed transcriptomic, clinical, and genomic data from TCGA database, and identified metabolism-related long non-coding RNAs, including 38 metabolic pathways [167]. They determined six clusters (N1-N6) and two tumor types (C1 and C2) of GC with different molecular profiles and responses to immunotherapy and chemotherapy.

Type C1 GC displayed higher immune and stromal scores and lower tumor purity than C2 GC. Additionally, it had greater infiltration by B cells, CD8+ T cells, dendritic cells, macrophages, and mast cells. This subtype was enriched in pathways related to interferon-gamma, interferon-alpha, and inflammatory responses. Conversely, the subtype C2 demonstrated enrichment in pancreas beta cells and bile acid metabolism pathways. Regarding genetic alterations, C1 GC presented mutations in *ARID1A*, *AHNAK2*, *PIK3CA*, and *ZBTB20*, while C2 GC had *TP53* mutations. Each subtype featured genes whose alterations influenced prognosis. Intriguingly, the alteration of *ABCA13* had opposing effects in subtypes C1 and C2. Concerning the treatment response, C1 GC exhibited greater sensitivity to gemcitabine, while C2 GC responded more favorably to atezolizumab, among other molecules.

### 3.13. Lin et al. (2021): Immune-Based Classification [168]

**Figure 9:**
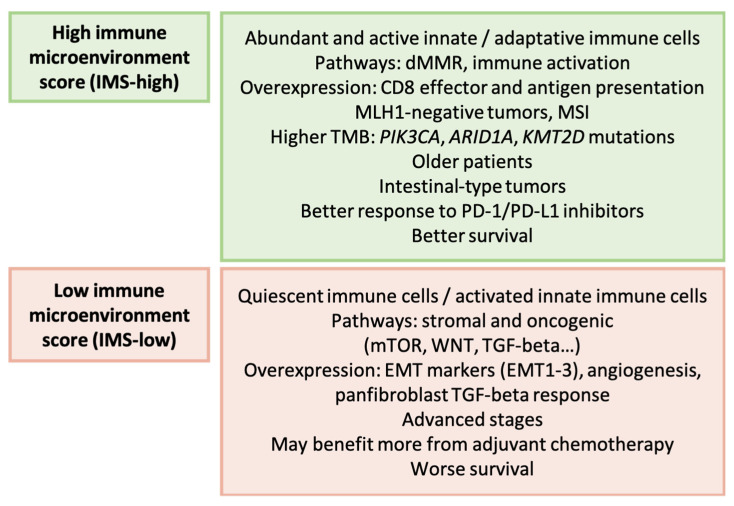
Lin’s classification. Clinicopathological and molecular features of GC types [168]. dMMR: deficient mismatch repair; MSI: microsatellite instability; TMB: tumor mutational burden; EMT: epithelial-mesenchymal transition.

In this study, the authors investigated six cohorts of GC patients and assessed the immune enrichment of tumors using 51 tumor microenvironment cell signatures from previously published research [168]. Subsequently, they developed an immune microenvironment score (IMS) and categorized GC into high IMS and low IMS groups. The main characteristics of these subtypes are presented in Figure 9. The high IMS subtype demonstrated enrichment in the MSI type of the ACRG, followed by the *TP53*-inactive group and the *TP53*-active group. In addition, IMS was higher in the EBV and MSI subtypes, according to TCGA. The authors found no differences in somatic copy number variations between the subtypes, but identified drugs with potential therapeutic effects on each subgroup.

### 3.14. Wu et al. (2022): Immune-Based Classification [169]

**Figure 10:**
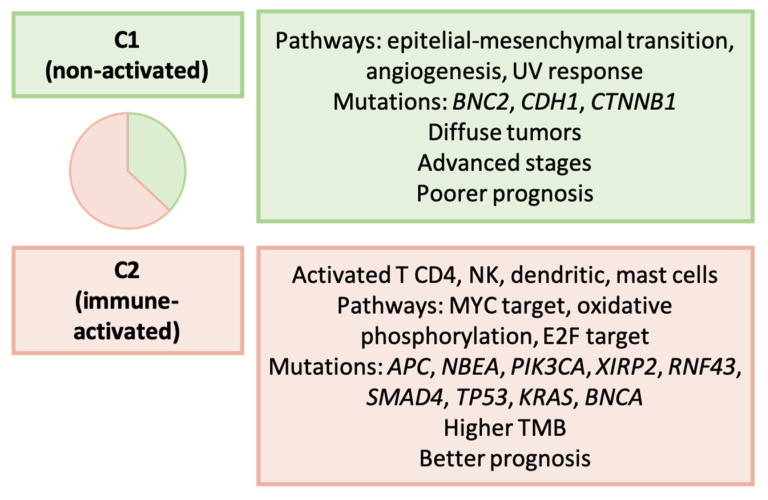
Wu’s classification. Clinicopathological and molecular characteristics of GC types [169]. TMB: tumor mutational burden.

In this investigation, Wu et al. examined 1386 samples sourced from three databases (TCGA, GEO, and an internal cohort), with TCGA data serving as the discovery set [169]. They identified two molecular subtypes of GC based on the immune environment, labeled as C1 (37%, “non-activated”) and C2 (63%, “immune-activated”), utilizing a panel of 390 immune-related genes. Figure 10 outlines the key molecular and clinicopathological features of each subtype. These subtypes exhibited varying associations with TCGA and the ACRG molecular groups. Notably, C1 GC was significantly enriched in the EMT type, according to the ACRG. Survival analysis revealed that this classification served as an independent prognosticator in GC, with C1 tumors being diagnosed at more advanced stages and having a worse prognosis.

### 3.15. Li et al. (2016): Tumor Mutational Burden [170]

In their study, Li et al. examined somatic mutations and clinical features across five geographically distinct cohorts [170]. Employing unsupervised clustering based on the somatic mutation count, the authors divided GC into the two following groups: regular (86.8%) and hypermutated (13.2%), with the latter being notably enriched in MSI tumors. The regular type displayed a distinctive APOBEC mutational pattern [55]. Moreover, regular GC showed alterations in pathways associated with genotoxic-oncogenic stress responses, histone modification/chromatin remodeling, growth factor receptor signaling, and Wnt signaling, presenting multiple molecular changes with therapeutic potential.

Further subtyping of the regular GC revealed two subgroups as follows: C1, with mutations in *TP53*, *XIRP2*, *APC*, *ERBB4*, and *AKAP6*; and C2, with mutations in *ARID1A*, *CDH1*, *PIK3CA*, and *RHOA*. The C2 cases were enriched in diffuse-type GC, tumors of the gastric body, exhibited worse prognosis, and were mainly classified as GS and CIN GC, according to TCGA. C1 GC cases were predominantly CIN tumors, according to TCGA. The classification of tumors into C1 and C2 proved to be an independent prognostic factor. Additionally, *CDH1* mutation was an independent prognosticator in diffuse-type GC.

### 3.16. Wei et al. (2022): Tumor Mutational Burden [171]

In this study, researchers analyzed genetic data, gene expression profiles, and clinical information from 433 patients in the TCGA database, and validated their findings in 433 additional cases from the GEO database [171].

**Figure 11:**
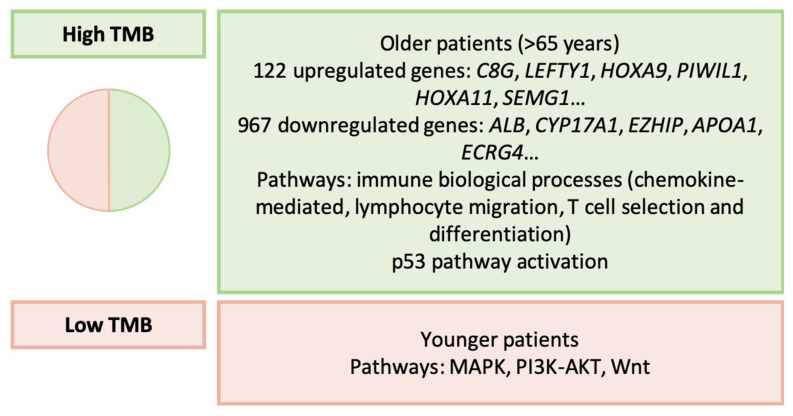
Wei’s classification. Clinical and molecular characteristics of GC types [171]. TMB: tumor mutational burden.

Initially, they determined two types of GC based on the TMB as follows: high TMB (49.9%) and low TMB (50.1%). The main characteristics of these subtypes are summarized in Figure 11. Subsequently, the authors developed a risk score that included the following four genes: *MATN3*, *UPK1B*, *GPX3*, and *RGS2*. They observed that high-risk patients had worse OS and DFS, higher stromal scores, high expression of immune checkpoints, and more immune cell infiltration. High-risk GC also exhibited increased sensitivity to gefitinib, vinorelbine, and gemcitabine. Using their risk score, they created a nomogram that, along with age and tumor stage, successfully stratified patients according to their OS and DFS. Finally, they developed a molecular classification based on differentially expressed genes related to TMB, dividing patients into three groups as follows: C1, C2, and C3. C2 GC showed reduced stromal and immune scores, increased tumor purity, low expression of immune checkpoints, and lower levels of immune cell infiltration. The C3 type was the least sensitive to gefitinib, gemcitabine, and sorafenib.

### 3.17. Weng et al. (2023): Epigenetic-Based Classification [172]

**Figure 12:**
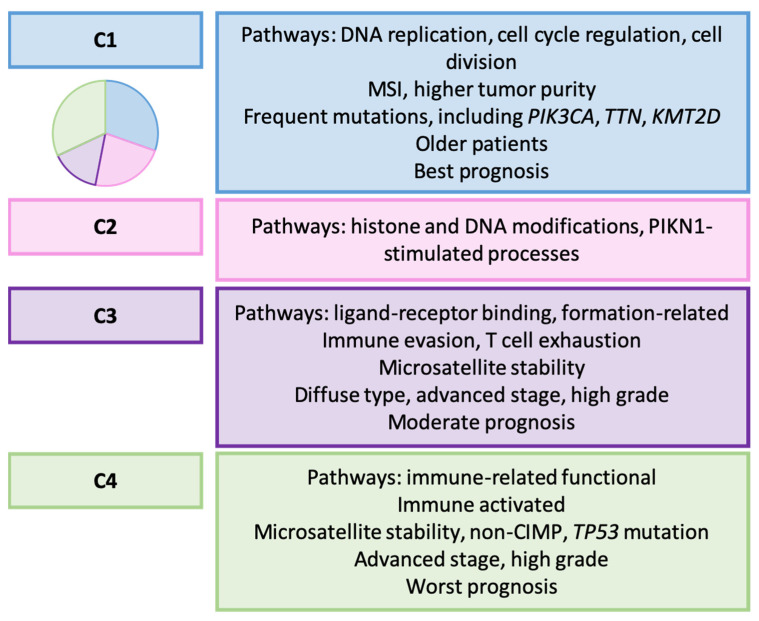
Weng’s classification. Clinicopathological and molecular features of GC types [172]. MSI: microsatellite instability; CIMP: CpG island methylator phenotype.

In their investigation, Weng et al. analyzed data from 1521 GC samples across five independent datasets from GEO and TCGA databases [172]. Utilizing an integrated clustering algorithm that combined miRNA expression and DNA methylation profiles, they identified four distinct molecular types of GC, subsequently validated into four independent multicenter cohorts as follows: cluster 1 (C1, 30.4%), C2 (22.7%), C3 (15%), and C4 (32%). Key characteristics of each subgroup are outlined in Figure 12.

Concerning their association with TCGA classification, types C1 and C2 were linked to MSI GC, C3 to GS, and C4 to CIN. Furthermore, their classification showed a correlation with the treatment response. C1 GC exhibited greater sensitivity to methotrexate, 5-FU, and paclitaxel. Patients in C2 benefited from apatinib and cisplatin treatment, and displayed potential sensitivity to warfarin. Conversely, C3 and C4 types demonstrated increased sensitivity to dasatinib and LY2606368, respectively. Intriguingly, the C3 subtype showed heightened resistance to traditional 5-FU, paclitaxel, apatinib, and/or cisplatin therapies.

### 3.18. Li et al. (2022): Proteomic-Based Classification [173]

Molecular classifications of GC have also been explored through the lens of the proteome. In a previous study, Li et al. developed a proteome-based classification of diffuse GC, dividing it into PX1–3 types. They observed that PX3 GC had a worse prognosis and a greater resistance to chemotherapy [174].

Subsequently, they investigated the response to neoadjuvant therapy in GC, conducting a comprehensive proteomic analysis of 206 tumor samples from therapy-naïve patients [173]. They established four GC subtypes as follows: G-I (14.1%), G-II (29.1%), G-III (47.1%), and G-IV (9.7%). The G-IV type demonstrated the poorest prognosis, with resistance to chemotherapy and anti-HER2 drugs escalating from G-I to G-IV. Pathway enrichment revealed the following distinctive features for each subtype: G-I was characterized by endocytosis-related proteins, G-II by glycolysis (gluconeogenesis and pantothenate/CoA biosynthesis pathways), G-III by lysosomal acid hydrolases and synthesized lysosomal enzymes, and G-IV by extracellular matrix-receptor interaction, focal adhesion, complement/coagulation cascades, and the PI3K-AKT signaling pathway. Moreover, G-IV was enriched in extracellular matrix proteins and displayed the fewest copy number variants.

The authors noted a correlation between their classification and the ACRG subtypes. G-IV tumors were predominantly mesenchymal-like with a lower frequency of MSI, while G-II GC exhibited higher MSI and hypermutation status. In terms of immune infiltration, G-IV tumors displayed fewer cytokines and antigen presentation, more monocytes, and fewer differentiated M1 macrophages. Lastly, G-II tumors had the highest percentage of *HER2*-amplified patients.

### 3.19. Tanaka et al. (2021): Ascites-Disseminated GC [175]

Tanaka et al. developed a molecular classification specific to ascites-disseminated GC [175]. They conducted a comprehensive study involving whole-genome sequencing, RNA sequencing, DNA methylation, and enhanced landscape analysis on tumor cells and cell lines from the malignant ascitic fluid samples of 98 patients.

Through gene expression profiling, the researchers identified two distinct molecular types within ascites-disseminated GC. One exhibited active super enhancers at the *ELF3*, *KLF5*, and *EHF* loci (non-EMT), while the other demonstrated TGF-beta activation through *SMAD3* and *TEF-1* (EMT). The EMT group, associated with EMT pathways, displayed a lower number of somatic mutations, *MET* amplification, *CDKN2A/B* homozygous loss, and the activation of the Hippo pathway involving components like *TEAD1*, *YAP1*, or *SMAD3*. Conversely, the non-EMT group exhibited *FGFR2* amplification.

Regarding the potential therapeutic implications of this classification, the authors noted that inhibitors targeting *FGFR2*, *MET*, *EGFR*, and *ALK* were effective in treating cell lines with alterations in these genes (amplification or fusion). Within the EMT subgroup, the inhibition of *MET* or the TEAD pathway could be particularly beneficial.

## 4. Clinical Impact of Molecular Classifications

### 4.1. Application of Molecular Classifications through Surrogate Markers

The integration of molecular classifications into clinical practice faces several challenges, including the limited availability of molecular techniques in many institutions, their high cost, the absence of optimal markers for diagnosing each molecular type, and their complexity. It should be noted that most research studies have relied on integrating vast amounts of data through complex computational techniques. Regarding technological availability, among the molecular techniques mentioned previously that have transformed the study of cancer biology, NGS is the most widely implemented platform in clinical practice, but still remains unavailable in some healthcare centers.

In contrast, IHC methods are readily available in all pathology laboratories, are more cost-effective, faster, and simpler to interpret [176]. IHC relies on antigen–antibody interactions to detect and highlight specific proteins on tissue slides, and is extensively used in daily practice to establish diagnosis, prognosis, or determine predictive markers in various pathologies [177,178]. Moreover, IHC generally requires smaller samples than molecular studies, allowing for the determination of the staining location at the subcellular level, the assessment of tumor heterogeneity, the quantification of the number of stained cells, and the intensity of staining. Breast cancer serves as a prime example of the successful implementation of molecular categories using IHC markers [179]. In situ hybridization techniques, which are part of molecular techniques, share many similarities with IHC and can complement IHC methods in the search for the optimal surrogate markers of molecular classifications [180]. For instance, in various types of tumors, HER2 IHC serves as a screening method to conduct *HER2* in situ hybridization in selected cases [97,181].

In GC, several studies have explored suitable surrogate markers for implementing TCGA and the ACRG classifications in clinical practice [182,183,184,185,186,187]. These investigations have spanned both Asian and Western populations, primarily utilizing IHC for mismatch repair proteins (MSH2, MSH6, PMS2, MLH1), p53, E-cadherin, or p21, along with simple molecular techniques like EBV fluorescence in situ hybridization.

In most cases, surrogate classifications have demonstrated prognostic value, although some studies have reported conflicting results [186]. Our research group developed an IHC classification based on the ACRG molecular classification, showing independent prognostic value in our GC patient cohort [188].

For the optimal application of these classifications in clinical practice, a consensus on the markers to use and the interpretation criteria is crucial, especially concerning p53 IHC. Several studies have considered the loss of p53 expression as a mutated pattern, while others focus on marker overexpression. Furthermore, some authors consider both the loss of expression and overexpression as mutated patterns. The cutoff point for p53 overexpression ranges from 20% to 70%, with 70% being the most common. Additionally, the assessment criteria for E-cadherin also vary, with some researchers considering the loss of membranous staining as an altered pattern, while others establish an expression cutoff point or label any tumor without strong and complete membrane staining as altered.

Interestingly, as far as we know, no studies have been published seeking surrogate markers for applying other molecular classifications beyond TCGA and the ACRG.

### 4.2. Equivalencies between Classifications, Prognostic, and Therapeutic Value

The main features of the molecular classifications of GC reviewed in this study are summarized in Table 3.

While these classifications, along with numerous others in the literature, may not be entirely equivalent, certain molecular categories exhibit similarities. For example, MSI-H tumors tend to occur in younger patients, belong to the intestinal type, and generally have a favorable prognosis. Hypermutated or immune-related tumors also typically show better prognostic outcomes, although some researchers have reported conflicting results. In any case, due to their high mutation rate, these tumors have a high neoantigen burden, thereby rendering immunotherapy potentially beneficial [189,190].

Tumors displaying EMT or those of the mesenchymal-like type are commonly associated with genomic stability, carry a poor prognosis, and are enriched in the diffuse type. Such tumors often exhibit greater resistance to traditional therapy, underscoring the importance of developing new drugs, particularly those targeting cell adhesion or angiogenesis-related pathways. Lastly, stable *TP53*-mutated GCs often display copy number variations and demonstrate a moderate to poor prognosis. However, these tumors tend to be more responsive to conventional chemotherapy [191].

It is worth noting that the presence of amplifications of tyrosine kinase receptor genes or cell cycle mediators, characteristic of stable *TP53*-mutated GC, along with other frequently observed alterations among GC subtypes such as *PIK3CA* mutation, holds potential therapeutic value.

## 5. Maximizing Impact through Interdisciplinary Collaboration

It is essential to recognize that the molecular studies routinely conducted for the clinical management of patients necessitate collaboration among multiple professionals, including biologists, mathematicians or computer scientists, pathology technicians, pathologists, and oncologists [192]. In our setting, biologists and/or pathology technicians are responsible for obtaining tissue slides and carrying out the molecular techniques. The pathologist confirms the presence of the tumor in the slides and quantifies tumor cellularity. Subsequently, molecular results are interpreted either by biologists or by pathologists. For complex techniques like NGS, the involvement of a computer scientist may also be necessary. On the other hand, the professional who best understands the indication of the study and the practical implications of the molecular result is the oncologist.

Thus, each molecular technique, similar to several other medical procedures, is the result of collaboration among diverse specialties. A distinguishing feature of molecular biology lies in the significant complexity and volume of information that must be managed to ensure a comprehensive understanding of results. This complexity is further compounded by the need for frequent updates, given the rapidly evolving nature of the discipline. To foster an environment of enrichment and maximize the utilization of available knowledge, most institutions conducting molecular techniques have established molecular boards, where the results are discussed with the participation of various professionals.

Nevertheless, there is still room for improvement in research. Firstly, efforts should concentrate on enhancing the training of all professionals who could potentially be involved in molecular pathology to encourage active participation in research studies. The literature reveals a notable gap between “basic” molecular studies, which address methodological aspects and results of large-scale molecular techniques, and “clinical” studies, which typically discuss therapeutic possibilities and ongoing clinical trials. However, a thorough understanding of all phases of the molecular procedure and its practical implications is indispensable for designing and executing research studies. Review articles that summarize molecular findings from a broader and more practical perspective, accessible to professionals from different specialties, provide valuable frameworks for understanding more specific studies within each discipline.

Secondly, improving communication among professionals within the context of molecular research is vital for refining the methodology and maximizing the practical impact of studies. In the realm of congresses and scientific sessions, those with the highest attendance are often specific to individual specialties, and focus on near-term practical applications. Unfortunately, participation from other specialties is usually limited. While guidelines from scientific societies tend to adopt a multidisciplinary approach, they may not be updated frequently and often prioritize clinical routines over research. Therefore, organizing regular interdisciplinary meetings on specific research topics involving clinicians and scientists would be highly beneficial. Consensus guidelines for future research studies could emerge from these meetings.

Thirdly, enhancing educational exchange between different specialties and among hospitals and research centers is advisable, both during the formal training period and in professional practice. The integration of diverse perspectives is becoming increasingly necessary, even from a practical standpoint.

In conclusion, professionals in molecular biology are confronted with vast amounts of information and complex technical tools. Facilitating interdisciplinary exchanges and fostering communication among specialists from different fields are crucial for effectively navigating through this wealth of information in order to guiding future studies towards practical applications.

## 6. Future Challenges

- Technological Advancements and Improvement of Novel Molecular Techniques:
  Novel molecular techniques beyond NGS or microarrays hold significant promise in GC. For instance, third-generation or SCS techniques can be valuable for characterizing GC in small samples, such as pre-surgical biopsies or liquid biopsy specimens, and for assessing intratumoral heterogeneity. These methods offer significant potential for analyzing the molecular profile of pre-neoadjuvant GC, monitoring patients, and identifying resistance mechanisms. Unfortunately, their broad availability or integration into clinical routine still requires significant progress.
- Validation of Molecular Classifications:
  Despite the publication of numerous molecular classifications in GC, those beyond TCGA and the ACRG have not been extensively validated. In addition, it is crucial to confirm previous results in geographically distinct cohorts due to the regional differences observed in the molecular, clinicopathological, and treatment features of GC. In this context, the promotion of open and collaborative GC databases could be beneficial.
- Identification of Surrogate Markers:
  Identifying optimal surrogate markers for applying TCGA and the ACRG classifications is essential, given the complex approaches used in these studies. Furthermore, exploring suitable surrogate markers for molecular classifications beyond those published by TCGA and the ACRG is also recommended, as this would address a significant gap in the GC literature.
- Consensus on Molecular Classifications:
  Given the heterogeneity of GC, achieving a consensus molecular classification is necessary for standardizing comparisons between studies in different cohorts. If complete consensus proves challenging, harmonizing the most consistent molecular types across classifications, such as MSI or high TMB tumors, those related to EMT, or those with *TP53* alterations, is advisable.
- Incorporation of Clinical and Histological Features:
  A notable correlation exists between molecular types and certain clinicopathological factors in GC, particularly the Laurén type, and, to a lesser extent, tumor location and other features. Integrating molecular alterations with histopathological findings that carry prognostic or therapeutic significance, akin to the methodologies applied in endometrial cancer, could prove to be a more effective strategy than presuming that a novel molecular classification will completely replace the value of histological features in GC. Achieving such an integrative classification relies on collaboration among different scientific disciplines and a holistic approach, involving all stakeholders in the context of GC diagnosis and treatment.
- Interdisciplinary Collaboration:
  Fostering an integrative approach necessitates collaboration among diverse professionals, encompassing biologists, clinicians, and pathologists. Essential to this is the training of clinicians and pathologists in molecular pathology, coupled with a deep understanding of clinical practice realities by basic researchers. Open forums for interdisciplinary discussions and knowledge exchanges remain vital for the successful translation of basic research into clinical practice.
- Improved Patient Stratification in Clinical Trials:
  Enhancing patient stratification in clinical trials could yield valuable insights into new biomarkers. For instance, studies have shown that categorizing patients according to the Laurén type enables the identification of distinct subgroups with diverse treatment responses [8]. The classification of patients in clinical trials based on molecular alterations or prominent histological factors, such as the Laurén type, would facilitate the individualized search for targeted therapies within more homogeneous groups, ultimately enhancing the precision and effectiveness of personalized approaches.

## 7. Conclusions

In the last decade, our understanding of the molecular landscape of cancer has led to the emergence of numerous molecular classifications across various tumor types. Some of these classifications, such as those for endometrial or breast cancer, have become integral components of routine clinical practices. Furthermore, the identification of molecular alterations as biomarkers for targeted therapy has revolutionized cancer treatment, improving patient outcomes and reducing the side effects associated with traditional oncologic approaches.

Despite these strides, technological progress has had a somewhat limited impact on patient prognosis and management in GC. In advanced tumors, therapeutic options beyond traditional chemotherapy are currently restricted to immunotherapy, antiangiogenic agents, and anti-HER2 drugs. Key advancements in molecular pathology for GC include the release of molecular classifications by TCGA and the ACRG. These groups conducted the comprehensive analyses of large GC cohorts, defining four similar yet not equivalent molecular subtypes. Additionally, various researchers have proposed diverse molecular classifications, highlighting substantial molecular heterogeneity within GC. Despite the differences among these classifications, some similarities exist, particularly in tumors with MSI, immune infiltration or activation, *TP53* mutation, or mesenchymal-like features.

A significant challenge in implementing these systems in real-world clinical settings lies in their complexity. In this context, the utilization of IHC markers as substitutes for TCGA or ACRG systems in previous studies has yielded promising results. This methodology provides a practical and cost-effective approach to the molecular classification of GC, despite variations in the selected markers and their assessment across studies.

In conclusion, for molecular classifications to exert a substantial impact on patient management and outcomes in GC, efforts must be directed towards establishing a consensus framework with categories that optimally influence prognosis and treatment selection. This entails validating different classifications in diverse cohorts, standardizing molecular subgroups, identifying optimal surrogate markers for practical application, integrating clinical, histological and molecular criteria, and fostering interdisciplinary collaboration. Additionally, patient stratification in clinical trials based on the molecular characteristics of GC may prove instrumental in developing more effective therapies, given the significant differences in tumor behavior and biology observed among the various molecular categories.

## Figures and Tables

**Table 1:** Shah’s classification. Main differential features between GC subtypes.

Subtypes	Upregulated	Downregulated
Proximal non-diffuse (vs. diffuse)	*TRIM32, PRF1, CXCL9, CXCL10, IF144L, PLA2G2A*	*PSCA, PGA3, XIST, SST, ABCA8*
Proximal non-diffuse (vs. distal non-diffuse)	*PF4V1, HMBO1, CYP2J2, DSC3, S100A12*	*MSLN, IGJ, ENPP4, PLA2G2A*
Diffuse (vs. distal non-diffuse)	*ABCA8, HMBOX1, COCH, S100A12, CYP2J2*	*IFI44L, HOXA9, MSLN, ENPP4*

**Table 2:** Ye’s classification. Main clinicopathological, immune, and molecular features of GC types.

Ye et al.	Molecular Features	Immune and Clinicopathological Features
Immune—suppressed (C1)	Pathways: *HIPPO, WNT, NOTCH*, cell cycle	Neutrophil-induced immune suppression Intestinal/tubular tumors No MSI ^a^
Metabolic (C2)	Pathways: *MYC, NRF2*	Abundance of T cells (Th1, cytotoxic, Tcm), B cells and dendritic cells
Mesenchymal—immune exhausted (C3)	Pathways: TGF-β, angiogenesis Enhanced EMT ^b^	Diffuse/poorly cohesive tumors Lower HER2 expression Worse prognosis
Hypermutated (C4)	Pathways: cell cycle, *TP53*, *PI3K* MSI CIMP ^c^	Abundance of Th2 cells EBV ^d^-related tumors Intestinal/tubular tumors Response to immunotherapy Better prognosis

^a^ MSI: microsatellite instability; ^b^ EMT: epithelial-mesenchymal transition; ^c^ CIMP: CpG island methylator phenotype; ^d^ EBV: Epstein–Barr virus.

**Table 3:** Molecular types of gastric cancer.

Authors	Year	Molecular Categories
TCGA ^a^ [42]	2014	MSI ^b^, genomically stable, EBV ^c^ positive, chromosomal instability
ACRG ^d^ [43]	2015	MSI, mesenchymal-like, TP53 active, TP53 inactive
Tan et al. [155]	2011	Intrinsic subtypes: G-INT and G-DIF
Lei et al. [156]	2013	Mesenchymal, proliferative, metabolic
Wang et al. [157]	2021	Subtypes 1–4
Shah et al. [158]	2011	Proximal non-diffuse, diffuse, distal non-diffuse
Furukawa et al. [161]	2022	Hypermutators, T-cell inflamed, EMT ^e^-high, EMT-low
Wang et al. [162]	2014	MSI, stable associated with EBV, stable non-EBV
Cheong et al. [164]	2022	Groups 1–4
Zhou et al. [165]	2023	Clusters 1–3 (functional status-based)
Ye et al. [156]	2022	Immune-suppressed (C1), metabolic (C2), mesenchymal-immune exhausted (C3), hypermutated (C4)
Li et al. [167]	2021	C1 and C2 types (metabolism-based)
Lin et al. [168]	2021	High and low immune microenvironment score
Wu et al. [169]	2022	Non-activated (C1) and immune activated (C2)
Li et al. [170]	2016	Regular, hypermutated
Wei et al. [171]	2022	High TMB ^f^, low TMB
Weng et al. [172]	2023	C1-C4 types (epigenetic-based)
Li et al. [174]	2022	PX1-3 types (proteomic-based)
Tanaka et al. [175]	2021	Two types of ascited-disseminated gastric cancer

^a^ TCGA: The Cancer Genome Atlas; ^b^ MSI: Microsatellite instability; ^c^ EBV: Epstein–Barr virus; ^d^ ACRG: Asian Cancer Research Group; ^e^ EMT: epithelial-mesenchymal transition; ^f^ TMB: tumor mutational burden.

## Data Availability

Literature review: all data were obtained from publicly available sources and referenced accordingly. No additional data sets were generated or analyzed for this review.
